# Biomarkers in osteoarthritis: current status and outlook — the FNIH Biomarkers Consortium PROGRESS OA study

**DOI:** 10.1007/s00256-023-04284-w

**Published:** 2023-01-24

**Authors:** David J. Hunter, Jamie E. Collins, Leticia Deveza, Steven C. Hoffmann, Virginia B. Kraus

**Affiliations:** 1 Sydney Musculoskeletal Health, Kolling Institute, Faculty of Medicine, University of Sydney, Australia and Rheumatology Department, Royal North Shore Hospital, St Leonards, NSW 2065 Australia; 2 Brigham and Women’s Hospital, Harvard Medical School, Boston, MA USA; 3 Foundation for the National Institutes of Health, Bethesda, North, MD USA; 4 Duke Molecular Physiology Institute, and Department of Medicine|, Duke University, Durham, NC USA

**Keywords:** Osteoarthritis, Imaging, Biomarkers

## Abstract

Currently, no disease-modifying therapies are approved for osteoarthritis (OA) use. One obstacle to trial success in this field has been our existing endpoints’ limited validity and responsiveness. To overcome this impasse, the Foundation for the NIH OA Biomarkers Consortium is focused on investigating biomarkers for a prognostic context of use for subsequent qualification through regulatory agencies. This narrative review describes this activity and the work underway, focusing on the PROGRESS OA study.

## Introduction

Osteoarthritis (OA) is a highly prevalent, disabling disease with a tremendous individual and societal burden [1]. Recent estimates suggest that 500 million people worldwide are affected by OA [2]. The risk of mobility disability (defined as needing help walking or climbing stairs) attributable to knee OA alone is more significant than that attributable to any other medical condition in people aged 65 years and older [3, 4].

Unfortunately for OA, there is no equivalent to glucose tolerance, quantifying lipid levels, and severity of atherosclerosis or hypertension, as is done to inform the detection and pre-emptive treatment of diabetes and cardiovascular disease, before the associated processes lead to end-organ failure [5]. In addition, even if we had such a biomarker, there are no therapies that have been approved by regulators to reduce the risk of OA progression [6]. However, a few drugs have recently been shown to beneficially modify structural progression in clinical trials [7, 8].

For an impact to be made for the millions living with chronic pain and disability of OA, a significant shift in the focus of OA research is critically needed to overcome barriers to the development of disease modifying pharmacological treatments. Biomarkers enhance the success of every phase in the drug development process; they increase the frequency of successful phase transitions (chances of a drug candidate advancing to the next phase of development) [9]. Two in four drugs fail in Phase 3 trials without biomarkers, whereas only one in four drug development programs fails with selection biomarkers [9].

Regulatory guidance describes a process for drug approval for specific indications in OA, including treatment of symptoms, delays in structural progression, and prevention of OA [10]. Radiographic joint space narrowing (JSN) is currently recommended by both the Federal Drug Administration (FDA) and European Medicines Agency (EMA) guidance documents as the imaging endpoint for clinical trials of disease-modifying OA drugs (DMOADs) [11]. From joint space narrowing (JSN) outcomes, the health, integrity and thickness of hyaline articular cartilage are inferred [12, 13].

If the currently recommended endpoint is chosen, namely JSN, the clinical trial would have to have a vast sample size followed for at least 2–3 years to demonstrate a significant incremental benefit of a novel therapy over and above that provided by currently available treatments [5]. The direct costs of conducting such trials and the overall duration of the therapeutic development and regulatory review process have dampened enthusiasm for the development of therapeutic agents in this area and have rendered the advancement of some novel treatments prohibitively expensive.

On the other hand, different and more efficient means of establishing the benefit of new drugs exist, offering the promise of timely access to new therapies. There is potentially tremendous value to health to accelerate the discovery and development processes for OA therapeutics through smaller, shorter, and more efficient studies, using validated endpoints other than radiographic JSN. The PROGRESS OA project intends to accelerate the qualification of novel biomarkers using JSN (predictive validity and responsiveness) as the standard metric benchmark to assess the relative success of other biomarkers.

**Fig. 1:**
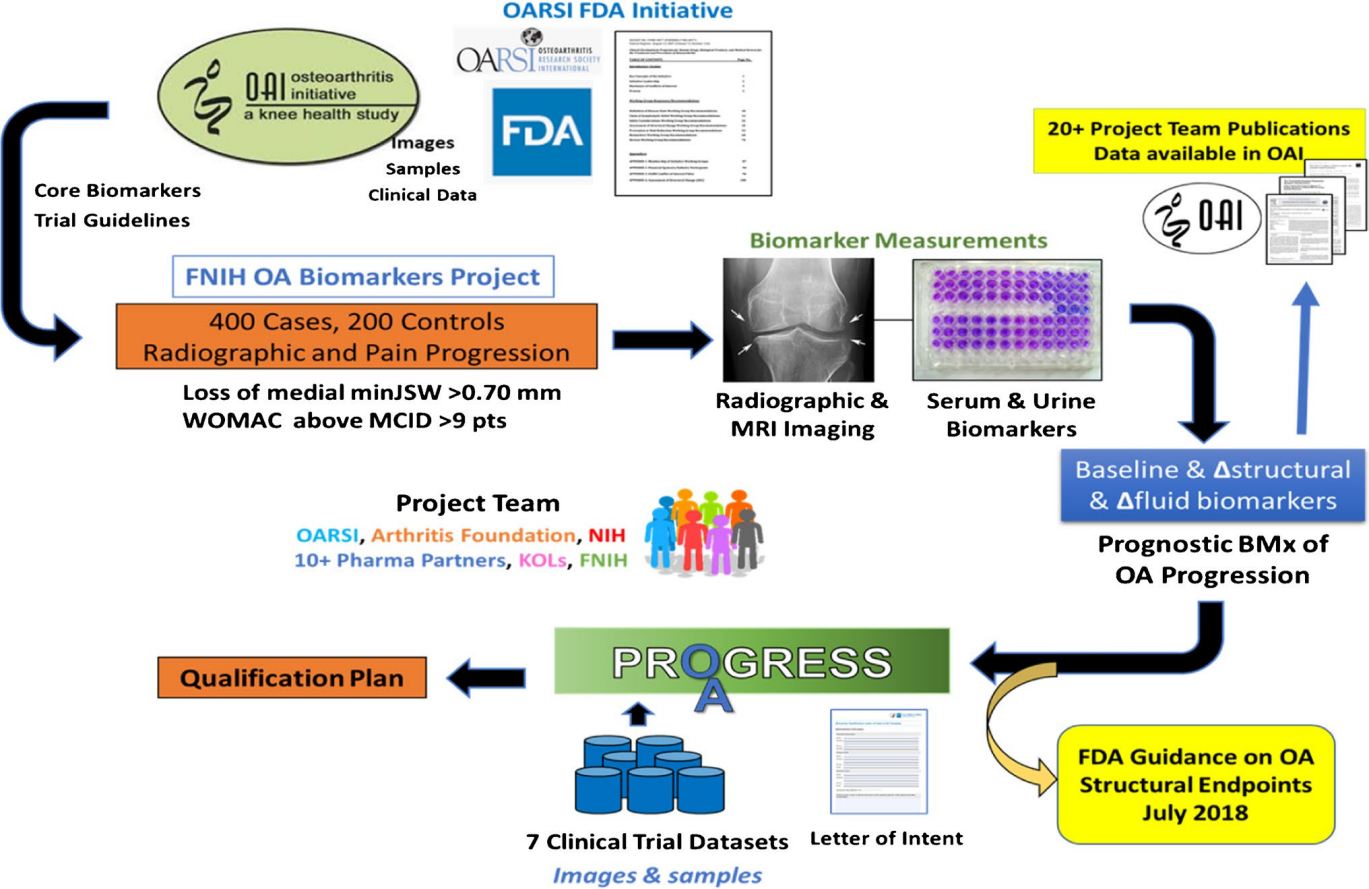
Project flow for the osteoarthritis (OA) biomarker project. BL, baseline; FDA, US Food and Drug Administration; FNIH, Foundation for the National Institutes of Health; KOLs, key opinion leaders; MCID, minimal clinically important difference; minJSW, minimal joint space width; MRI, magnetic resonance imaging; NIH, National Institutes of Health; OAI, Osteoarthritis Initiative; OARSI, Osteoarthritis Research Society International; WOMAC, Western Ontario and McMaster Universities. From [15]

In addition to biochemical markers, in OA, further refinement and improvement of measures of joint structural change are also needed to overcome the limited responsiveness of existing imaging biomarkers, such as the poor relation in individual patients between joint structural pathology (e.g., joint space narrowing on radiographs) and symptomatic disease [5]. To overcome these obstacles, the FNIH OA Biomarkers Consortium (FNIH BC) undertook an extensive Phase 1 biomarker qualification study from 2012 to 2015 using a nested case-control sample of progressive knee OA within the Osteoarthritis Initiative (OAI) [14]. The overarching project objective was to establish the predictive validity of disease progression biomarkers and assess the responsiveness of several imaging and biochemical markers pertinent to knee OA. The results of this study are complete, and we are now pursuing Phase 2 qualification of the biomarkers in independent cohorts and existing completed clinical trials. Figure 1 illustrates the foundational resources that support the FNIH BC OA Biomarkers and PROGRESS OA projects and the strategic involvement of the multisector consortium guiding the projects and field toward the validation and regulatory consideration of select biomarkers of risk for OA progression [15].

The primary objective of the PROGRESS OA study is to assess the prognostic capacity of imaging and/or biochemical biomarkers measured at baseline in predicting disease progression. Our core hypothesis is that a single (or combinatorial) imaging and/ or biochemical biomarker(s) can predict OA disease progression. Our objective is to pursue the qualification of biomarkers pertinent to knee OA for a prognostic context of use (COU): baseline predicting disease progression and short-term change predicting change over longer-term in OA using the placebo group arms of multiple completed OA clinical trials. Further details on PROGRESS OA are available from the submission to the FDA [16].

Our deployment of novel biomarker measures in existing longitudinal and clinical trials will enable us to determine the prognostic capacity of these biomarkers. This paper will describe the background results from the first phase of our study, the design of the Phase 2 PROGRESS OA study, including the proposed analyses, steps toward FDA qualification, other activities to facilitate qualification occurring in the OA community, and the next steps.

### Background results from Phase 1 FNIH study

Phase 1 of the FNIH study has shown the prognostic validity of several biochemical and imaging markers in the Osteoarthritis Initiative cohort. The results of this study were described in detail in previous publications [17–22]. In summary, using a nested case-control design, 600 knees were allocated into one of four groups pre-defined based on progression status at 48 months: pain progression only, radiographic progression only, pain and radiographic progression (primary outcome), or no progression either pain or radiographic. Biomarkers included MRI (quantitative (Q) cartilage thickness and volume, semi-quantitative (SQ) MRI markers, bone shape and area, Q meniscal volume) [19], radiographic (trabecular bone texture (TBT)) [21], and serum and/or urine biochemical markers [20]. Biomarkers were analyzed individually and in a multivariable models [22] to determine the optimal combination of biomarkers that could provide prognostic utility in OA disease-modifying trials. Three step-wise selection methods were tested in multivariable logistic regression models (complex vs. parsimonious models).

The baseline markers with the best performance in multivariable models to predict pain and radiographic progression were semi-quantitative osteophytes and Hoffa-synovitis and quantitative cartilage thickness (central medial femoral and central lateral femoral) and patella shape with C-statistics ranging from 0.641 to 0.671 in the different models (Table 1) [22]. In the analysis using 24-month change in biomarkers to predict pain and radiographic progression at 48 months, different biomarkers were determined to have the best performance, including semi-quantitative (effusion-synovitis, meniscal, and cartilage morphology) and quantitative measures of cartilage thickness and volume, radiographic TBT, and urinary NTX-I (C-statistics 0.680–0.724) (Table 2). The secondary analysis focused on radiographic progression only (regardless of pain progression status) and selected a different combination of biomarkers (baseline and 24-month change) with higher C-statistics (0.716–0.832).

**Table 1:** Multivariable modeling — baseline biomarker to predict radiographic and pain progression (*n*=550) [22]

	Imaging biomarkers only			Imaging + biochemical biomarkers		
MODEL	M1	M2	M3*	M4	M5**	M6*
Selection Method	Stepwise, AIC	Stepwise, SBC	Stepwise, *p*-value	Stepwise, AIC	Stepwise, SBC	Stepwise, *p*-value
Model characteristics						
AUC (unadjusted)	0.684	0.646	0.684	0.688	0.646	0.684
AUC (adjusted)***	0.718	0.688	0.718	0.722	0.688	0.718
AUC (adjusted, 10 fold cross-validation)	0.669	0.641	0.669	0.671	0.641	0.669
IDI	0.0832	0.0548	0.0832	0.0861	0.0548	0.0832
NRI	0.4746	0.4048	0.4746	0.5007	0.4048	0.4746
%cases correctly reclassified^1^	28%	29%	28%	28%	29%	28%
%controls correctly reclassified^2^	19%	12%	19%	22%	12%	19%
Biomarkers included						
Semi-quantitative MRI	Hoffa-synovitis Number of locations affected by any osteophyte (semi-quantitative)	Number of locations affected by any osteophyte (semi-quantitative)	Hoffa-synovitis Number of locations affected by any osteophyte (semi-quantitative)	Hoffa-synovitis Number of locations affected by any osteophyte (semi-quantitative)	Number of locations affected by any osteophyte (semi-quantitative)	Hoffa-synovitis Number of locations affected by any osteophyte (semi-quantitative)
Quantitative cartilage thickness	Quantitative mean cartilage thickness - central lateral femur (internal)		Quantitative mean cartilage thickness - central lateral femur (internal)	Quantitative mean cartilage thickness - central lateral femur (internal)		Quantitative mean cartilage thickness - central lateral femur (internal)
	Quantitative mean cartilage thickness - central medial femur (external)		Quantitative mean cartilage thickness - central medial femur (external)	Quantitative mean cartilage thickness - central medial femur (external)		Quantitative mean cartilage thickness - central medial femur (external)
Bone shape on MRI	Patella shape	Patella shape	Patella shape	Patella shape	Patella shape	Patella shape
Biochemical markers				Serum NTX-I ****		

*AUC* the receiver operating characteristic (ROC) curve (C-statistic)

*IDI* the integrated discrimination Improvement

*NRI* the category-less net reclassification for each model: 1: %cases correctly reclassified = % of cases with a higher probability of being a case in new model vs. old;

2: %controls correctly reclassified = % of controls with a lower probability of being a case in new model vs. old;

Models 1-6 (M1-M6) as previously described [22] were as follows: Multivariable logistic regression models were built using three different step-wise selection methods (complex vs. parsimonious models). We first considered models with imaging parameters only (models 1 to 3) and then added the biochemical markers in a second step (models 4 to 6) in order to assess the additional prognostic value of adding biochemical markers to imaging parameters only. Three different stepwise selection methods were used to determine the best subset of predictors: (1) Akaike Information Criterion (AIC) (models 1 and 4); (2) Schwarz Bayesian Criterion (SBC) (models 2 and 5); and (3) *p*-value (models 3 and 6) (*p*=0.2 for entry/0.1 for retention)

*The biomarkers selected were the same as model 1

**The biomarkers selected were the same as model 2

***Adjusted for age, BMI, sex, race, baseline minimum joint space width, baseline WOMAC pain score, baseline KLG and use of pain medications

****Interpolated research value if the below lower limit of quantification

**Table 2:** Multivariable modeling — 24-month change in biomarkers to predict radiographic and pain progression (*n*=539) [22]

	Imaging biomarkers only			Imaging + biochemical biomarkers		
MODEL	M1	M2	M3	M4	M5	M6
Selection Method	Stepwise, AIC	Stepwise, SBC	Stepwise, *p*-value	Stepwise, AIC	Stepwise, SBC	Stepwise, *p*-value
Model characteristics						
AUC (unadjusted)	0.765	0.713	0.755	0.774	0.713	0.764
AUC (adjusted)*	0.777	0.730	0.770	0.788	0.731	0.781
AUC (adjusted, 10 fold cross-validation)*	0.713	0.680	0.713	0.724	0.683	0.724
IDI (vs. covariates only model)	0.1612	0.1129	0.1510	0.1699	0.1145	0.1616
NRI (vs. covariates only model)	0.6611	0.5164	0.6951	0.6498	0.5482	0.6819
%cases correctly reclassified^1^	23%	10%	26%	25%	15%	26%
%controls correctly reclassified^2^	43%	41%	43%	40%	40%	42%
**Biomarkers included**						
Semi-quantitative MRI	Worsening in effusion-synovitis	Worsening in effusion-synovitis	Worsening in effusion-synovitis	Worsening in effusion-synovitis	Worsening in effusion-synovitis	Worsening in effusion-synovitis
	Worsening in meniscal morphology	Worsening in meniscal morphology	Worsening in meniscal morphology	Worsening in meniscal morphology	Worsening in meniscal morphology	Worsening in meniscal morphology
	Increase in number of subregions with worsening cartilage morphology (thickness and surface area)		Increase in number of subregions with worsening cartilage morphology (thickness and surface area)	Increase in number of subregions with worsening cartilage morphology (thickness and surface area)		Increase in number of subregions with worsening cartilage morphology (thickness and surface area)
	Worsening in Hoffa-synovitis			Worsening in Hoffa-synovitis		
	No change in osteophyte score (vs. increase)			No change in osteophyte score (vs. increase)		
Quantitative cartilage thickness	Loss of cartilage thickness in the central medial femur (center)	Loss of cartilage thickness in the central medial femur (center)		Loss of cartilage thickness in the central medial femur (center)	Loss of cartilage thickness in the central medial femur (center)	
	Loss of cartilage thickness in the medial tibia		Loss of cartilage thickness in the medial tibia	Loss of cartilage thickness in the medial tibia		Loss of cartilage thickness in the medial tibia
Bone area on MRI	Change in lateral patellofemoral bone area		Change in lateral patellofemoral bone area	Change in lateral patellofemoral bone area		Change in lateral patellofemoral bone area
Biochemical marker				Increase in urine NTXI**	Increase in serum NTXI**	Increase in urine NTXI**
Trabecular bone texture	Intercept (Horizontal)	Intercept (Horizontal)	Intercept (Horizontal)	Intercept (Horizontal)		Intercept (Horizontal)
	Slope (Horizontal)	Slope (Horizontal)	Slope (Horizontal)	Slope (Horizontal)		Slope (Horizontal)

Time-integrated-concentrations used for biochemical and trabecular bone texture biomarkers.

*AUC* the receiver operating characteristic (ROC) curve (C-statistic);

*IDI* the integrated discrimination Improvement;

*NRI* the category-less net reclassification for each model: 1: %cases correctly reclassified = % of cases with a higher probability of being a case in new model vs. old;

2: %controls correctly reclassified = % of controls with a lower probability of being a case in new model vs. old

Models 1-6 (M1-M6) as previously described [22] were as follows: Multivariable logistic regression models were built using three different step-wise selection methods (complex vs. parsimonious models). We first considered models with imaging parameters only (models 1 to 3) and then added the biochemical markers in a second step (models 4 to 6) in order to assess the additional prognostic value of adding biochemical markers to imaging parameters only. Three different stepwise selection methods were used to determine the best subset of predictors: (1) Akaike Information Criterion (AIC) (models 1 and 4); (2) Schwarz Bayesian Criterion (SBC) (models 2 and 5); and (3) *p*-value (models 3 and 6) (*p*=0.2 for entry/0.1 for retention)

*Adjusted for age, BMI, sex, race, baseline minimum joint space width, baseline WOMAC pain score, baseline KLG and use of pain medications

**Interpolated research value if the below lower limit of quantification

### Phase 2 — PROGRESS OA study

#### Aims

The Phase 2 or PROGRESS OA FNIH Biomarkers Consortium project will utilize clinical data, MRI and radiographic images and biospecimens, to assess the following aims. Of note, these primary aims represent validation, in additional cohorts and trials, of biomarker capabilities demonstrated in Phase 1 of the OA FNIH study. Successful validation of any of these biomarkers in this Phase 2 OA FNIH study will, therefore, likely provide sufficient data for regulatory consideration for the qualification of a number of these biomarkers, which will be pursued subsequently. The aims are for a prognostic context of use [5, 23]:

(A) Validation of the ability of a baseline set of plain radiographic measures, MRI measures and biochemical markers to predict the likelihood of disease progression in subjects for qualification as *prognostic* markers with which to enrich OA trials for progressors. [=bl-PROG]
(B) Assess short-term change of a set of plain radiographic measures, MRI measures and biochemical markers to predict the likelihood of disease progression in placebo-treated subjects for qualification as prognostic markers to facilitate early identification of subjects likely to progress without treatment. [=change-PROG]

This will entail the following procedural steps:

1. Send high-quality biospecimens to selected assay vendors to evaluate the performance of up to 9 biochemical markers plus creatinine
2. Send MRI images to selected imaging processing groups to evaluate the performance of MRI markers
3. Send radiographic images to 1 selected imaging processing group and 1 fractal data processing group to evaluate the performance of the bone trabecular texture (TBT) imaging marker
4. Analyze the results of these imaging and biochemical data, singly and in combination
5. Review and approve the results of all analyses of the imaging and biochemical markers (by the Project Team) before being published in a suitable peer-reviewed journal(s)
6. Make the data from the analyses broadly and publicly available and promptly publish relevant results by the investigators in collaboration with the Project Teams
7. Pursue biomarker qualification with both FDA and EMA

#### Design

This project will utilize several existing clinical trial resources with existing biospecimens and imaging repositories to assess imaging and biochemical biomarkers for their ability to fulfill roles as prognostic markers (Table 3), defined as follows: prognostic biomarkers--baseline and/or short-term change predicting disease/JSW change over longer-term in the placebo groups of the trials. Notably, baseline prognostic biomarkers will provide a new way to enrich OA trials for subjects likely to progress; biomarkers qualified in this category could be employed in any number of the enrichment strategies for clinical trials proposed by the FDA [24]. Tables 3 and 4 summarize these categories as bl-PROG and change-PROG, respectively.

**Table 3:** Summary of proposed biomarkers for Phase 2 of the PROGRESS OA Project

Biomarker name (parameter type)	Timepoints	Treatment and/or placebo group tested	Levels of proposed qualification
Semi-quantitative analyses: bone marrow lesion size and location; synovitis and effusion; meniscal scoring; cartilage scoring; osteophyte scoring (MRI)	ALL	Placebo	Prog-BL, Prog-change
Total and central subregion cartilage volume (MRI)	ALL	Placebo	Prog-BL, Prog-change
TBT – bone trabecular texture (radiographic imaging parameters)	ALL	Placebo	Prog-BL, Prog-change
Biochemical: urinary (u) CTXII (biochemical), serum (s) HA, sNTXI, uC2C HUSA, sCTXI, sPIIANP, uCTXIalpha, uCTXIbeta, uNTXI, ucreatinine	ALL	Placebo	Prog-BL, Prog-change

Footnote: *bl*, PROG (baseline prognostic); *change*, PROG (change prognostic)

**Table 4:** Proposed biochemical markers for Phase 2 of the FNIH OA Biomarkers Consortium Project

Biomarker	Full name	Manufacturer (Cat. number)	Rationale	Results from Phase 1
uCTXII	C-terminal crosslinked telopeptide type II collagen	IDS (AC-10F1)	Multivariable modeling results	Associated with all three progressor groups; odds of predicting case status were stronger comparing cases to 'pure' non-progressors; baseline, 12M and 24M time-integrated concentration (TIC) predict primary status; every timepoint (baseline, 12M and 24M) predicts every progressor type in secondary analyses
sHA	hyaluronan	Corgenix (029-001)	Multivariable modeling results	Stronger odds of predicting case status when cases were compared to 'pure' non-progressors; 24M TIC predicts case status in primary analysis; 12M and 24M TIC predict case status in secondary analysis
sNTXI	N-telopeptide of type I collagen	ALERE -Osteomark (Inverness Medical) (9021)	Multivariable modeling results	12M and 24M TIC predict case status in primary and secondary analysis
uC2C-HUSA	C-terminal cleavage product of human type II collagen human urinary sandwich assay	IBEX (60-1017)	Univariable modeling results (unadjusted and adjusted)	Associated with all three progressor groups; odds of predicting case status were stronger comparing cases to 'pure' non-progressors; 24M TIC predicts primary status; every timepoint (except baseline for structural outcome) predicts every progressor type in secondary analyses
sCTXI	C-terminal crosslinked telopeptide of type I collagen	IDS (AC-02F1)	Univariable modeling results (unadjusted and adjusted)	Stronger odds of predicting case status when cases were compared to 'pure' non-progressors; 12M and 24M TIC predict case status in primary analysis; all timepoints predict case status in secondary analyses
sPIIBNP (pro-C2)	N-terminal propeptide of type IIB collagen	Nordic Bioscience (Herlev, Denmark)	Univariable modeling results (unadjusted and adjusted)	In two longitudinal cohorts (NYU and SMC), study participants with low PRO-C2 levels had greater joint space loss compared with subjects with high PRO-C2 corresponding to a 3.4 higher risk of knee OA progression
uCTXIα	Alpha isomerized versions of CTXI	IDS (AC-04F1)	Univariable modeling results (unadjusted and adjusted)	Stronger odds of predicting case status when cases were compared to 'pure' non-progressors; baseline, 12M and 24M TIC predict case status in primary analysis; 12M and 24M TIC predict case status in secondary analyses
uCTXIβ	beta isomerized version of CTXI	IDS (AC-05F1)	Univariable modeling results (unadjusted and adjusted)	Stronger odds of predicting case status when cases were compared to 'pure' non-progressors; 12M and 24M TIC predict case status in primary and secondary analyses
uNTXI	N-telopeptide of type I collagen	ALERE -Osteomark (Inverness Medical) (9006)	Univariable modeling results (unadjusted and adjusted)	Stronger odds of predicting case status when cases were compared to 'pure' non-progressors; 12M and 24M TIC predict case status in primary and secondary analyses
uCreatinine	Creatinine (for normalization of urinary markers)	Quidel MicroVue (8009)	To normalize urinary markers	

*u*, urinary; *s*, serum

As listed in Table 3, plain radiographic, MRI, and biochemical markers will be assessed in existing completed trials to measure the ability to predict clinical outcomes and their ability to change over time. A more detailed description of each of these measures follows below.

#### Imaging markers

Two major imaging methodologies (encompassing major MRI parameters and novel bone plain radiographic measures) will be assessed. MRI parameters will be assessed for the proposed contexts of use on the basis that they performed well in Phase 1 univariate and/or multivariable models that included both imaging and biochemical markers to predict longer-term clinical outcomes [22]. The novel bone plain radiographic measure (bone trabecular texture) will be assessed on baseline samples based on Phase 1 and strong historical performance [25] as a predictor of progression that could be used as a screening tool to enrich trials for progressors; secondary analysis includes evaluation of its change over time as a prognostic indicator. These MRI instruments have shown adequate reliability, specificity, and sensitivity, and the ability to detect lesion progression over 1–2 years. As described below and in the Phase 1 proposal, the bone trabecular texture (TBT) measure is also highly reliable [21, 26].

#### Semi-quantitative analyses

Semi-quantitative MRI scoring is a valuable method for performing a multi-feature assessment of the knee using conventional MRI acquisitions [27–30]. This method uses an observer-dependent semi-quantitative approach to score various articular features believed to be relevant to knee functional integrity and/or potentially involved in the pathophysiology of OA [31]. These features can include articular cartilage integrity, marginal and central osteophytes, subarticular bone marrow abnormality, subarticular cysts, subarticular bone attrition, synovitis/effusion, medial and lateral meniscal integrity, anterior and posterior cruciate ligament integrity, medial and lateral collateral ligament integrity, intra-articular loose bodies, and periarticular cysts/bursitis. These instruments for scoring OA on an MRI have shown adequate reliability, specificity and sensitivity, and the ability to detect lesion progression over 1–2 years (14, 15). Furthermore, baseline and change scores performed well in multivariable analyses from Phase 1 in predicting longer-term clinical outcomes [22].

#### Quantitative cartilage morphometry

MRI’s three-dimensional (3D) coverage of an entire cartilaginous region allows for the direct quantification of cartilage volume, surface areas, and thickness. Quantitative analysis of cartilage morphometry from MRI is becoming more widely used to assess OA [32]. Measurements of cartilage volume via MRI have been previously shown to correlate well with the ex vivo assessments of cartilage volume (stripped away from the bone) [31, 33, 34]. The measurement of cartilage volume or thickness provides quantitative data with which to monitor the progression of OA [35]. Annual changes in cartilage volume/thickness exceeded the precision errors and appear to be associated with clinical symptoms and with time to knee arthroplasty [36, 37]. One study detected cartilage volume loss without a change in radiographic joint space width, suggesting that MRI has greater sensitivity to change than radiograph [38]. These methods are described in more detail elsewhere [32, 39–41].

#### Radiographic parameters

Bone trabecular texture (TBT) represents the state of the vertical and horizontal trabeculae of a standardized region of interest of bone. TBT as a biomarker measure has been extensively validated and is an excellent predictor of structural progression [26, 42, 43]. Specifically, baseline TBT of the medial subchondral tibia in knee OA cohorts has been shown to predict radiographically and MRI-defined OA structural progression over the ensuing 12–36 months [26, 42, 44]. TBT also changes concurrently with loss of joint space width, joint space area, and MRI cartilage volume in knee OA progression [26, 42]. Although only a modest predictor of clinically relevant progression (structural AND pain worsening) as demonstrated in Phase I of the OA FNIH study, it is a strong predictor of structural radiographic progression in the Osteoarthritis Initiative and MOST cohorts [43, 44]. For this reason, and it is readily acquired from knee radiographs that are a standard part of clinical trial practice, it is deemed very useful for exploring at the Phase 2 level of this project.

Quantification of TBT is a two-step process. In the initial step, fractal signature analysis is performed on the tibial subchondral bone of the medial compartment of a knee radiograph using a semi-automated software designed initially by Optasia Medical (Manchester, UK) and deployed by Clario (Seattle, WA). In the second data reduction step, the fractal data are reduced to 6 parameters suitable for multivariable regression for evaluation of association with progression status or evaluation by cutoff scores for use as a screening tool to enrich OA trials for progressors. Automated software for performing this step has been developed at Duke University and will be deployed on the extracted fractal data. In addition, this imaging software generates minimum JSW (mJSW) and joint space area (JSA) and could be made available from this digital analysis. All TBT parameters, mJSW and JSA, are determined with high reliability and precision. The TBT analysis will be conducted on the baseline radiographs of the placebo arms of all appropriate studies to determine its predictive capability in this Phase 2 analysis. The Optasia software will also be used to quantify radiographic mJSW (e.g., at baseline and endpoint), for change in this metric over time to quantify, with high reliability, this progressor metric harmonized across all subjects of all the included trials, using the same tool.

#### Biochemical markers

The biomarkers chosen for the Phase 2 FNIH analyses all demonstrated the ability to predict clinically relevant progression, singly or in combination, in Phase 1 and with high reliability [20, 45]. The following biochemical markers will be performed on all available serum (s)/urine (u) samples based on promising results in the Phase 1 OA FNIH study [20] (Table 4 below):

*u*, urinary; *s*, serum

In the FNIH Phase 1 study, there were two main OA groups: “pure” non-progressors (neither pain nor joint space loss (JSL) worsening over 48 months), and clinically relevant progressor cases (with both pain and joint space loss (JSL) worsening over 48 months) defined as knee radiographic JSL progression as a decrease in minJSW >0.7mm from 24 to 48 months from baseline, and sustained pain worsening, defined as a WOMAC pain increase from 24 to 48 months of >9 units on a normalized 100 unit scale, sustained on at least two follow-up visits over 60 months from baseline. Both of these criteria are considered to be above a minimum clinically important differences as previously described [19]. In all, there were three progressor subgroups: JSL only, pain worsening only, pain worsening, and JSL.

#### List of clinical trials

We have obtained samples and images (MRI and radiographic) from completed clinical trials in OA to leverage their existing data to expedite the biomarker qualification process (summarized in Table 5). These trials test a range of therapeutic interventions whose treatment arms in the future could be leveraged to evaluate the treatment effects on these biomarkers as outcomes. The biological specimens and imaging resources collected in these datasets are unparalleled and provide a rich “real-world” clinical trial resource for further investigations such as that proposed here. More specifically, plain radiographic, MRI, and biochemical markers will be assessed to measure the ability to predict clinical outcomes and their ability to change over time. The images (radiographs and MRI) and biospecimens have already been acquired during these clinical trials, and these will be evaluated for the biomarkers described.

**Table 5:** Summary of relevant data available from these clinical trials

Trial Name	Agent (Caveats)	Sponsor	Prior publications	Trial duration	MRI images suitable	X-ray at follow up	Biospecimens available for assay	Estimated number of MRIs at baseline (placebo arm)	Estimated number of biospecimens at baseline (placebo arm)	Timepoints	Type of Biomarker Qualification Possible
Cindunistat (NCT00565812)	Cindunistat (small study)	Pfizer	Ann Rheum Dis. 2013 Feb;72(2):187-95.	24 months	Yes	Yes	No	27		MRI: 0, 6, 12 and 24 months	MRI: bl-PROG, change-PROG
Calcitonin (NCT00486434, NCT00704847)	Calcitonin (low resolution MRI)	Novartis	Osteoarthritis Cartilage. 2015 Apr;23(4):532-43.	24 months	No	Yes	Yes	-	792	Serum and urine: 0, 12 and 24 months	Serum and urine: bl-PROG; change-PROG
VIDEO (Arden) (ISRCTN94818153)	VIDEO-Vitamin D (lack of progression in the placebo group)	Oxford	Osteoarthritis Cartilage. 2016 Nov;24(11):1858-1866.	36 months	Yes	Yes	Yes	24	191	MRI: 0, 12, 24, 36 months Plasma, Serum, Urine: 0, 3, 6, 12, 24, 36 months	MRI: bl-PROG; change-PROG Serum and urine: bl-PROG; change-PROG
Strontium Ranelate (SEKOIA) (NCT02072070)	Strontium ranelate	Servier	Ann Rheum Dis. 2013;72(2):179-86	36 months	Yes	Yes	No	90		MRI: 0 and 36 months	MRI: bl-PROG;
ILLUSTRATE-K- Lutikizumab (Abbive) (NCT02087904)	Lutikizumab	Abbvie	Arthritis Rheumatol. 2019 Jul;71(7):1056-1069.	12 months	Yes	Yes	No	60	-	MRI: 0, 6 and 12 months	MRI: bl-PROG; change-PROG
ROCCELLA (Galapagos) (NCT03595618)	GLPG1972/S201086	Galapagos	Osteoarthritis and Cartilage Open, 3 (4). 100209. ISSN 2665-9131	12 months	Yes	Yes	No	232	-	MRI: 0, 6 and 12 months	MRI: bl-PROG; change-PROG

*sCTX (uCTXII and osteocalcin) already run on the CALCITONIN trial, so only 9 assays are required

The number of pain progressors, defined as an increase of ≥ 9 points on the WOMAC pain subscale (0–100 scale), and radiographic progressors (defined as JSN ≥ 0.7 mm over the follow-up period) in each trial are detailed in Table 6.

**Table 6:** Prevalence of progression by study

Trial Name	Sponsor	Progression JSL ≥ 0.7mm *n* (%)	Progression WOMAC pain ≥ 9 *n ***(%)**
Cindunistat (NCT00565812)	Pfizer	1 (5%)	5 (19%)
Calcitonin (NCT00486434)	Novartis	58 (12%)	120 (25%)
Calcitonin (NCT00704847)	Novartis	48 (14%)	91 (25%)
VIDEO (Arden) (ISRCTN94818153)	Oxford	13 (26%)	32 (63%)
Strontium Ranelate (SEKOIA) (NCT02072070)	Servier	17 (18%)	29 (31%)
ILLUSTRATE-K- Lutikizumab (Abbvie) (NCT02087904)	Abbvie	2 (3%)	15 (24%)
ROCCELLA (Galapagos) (NCT03595618)	Galapagos	26 (13%)	27 (13%)
Total		165 (13%)	319 (24%)

#### Cindunistat study (Pfizer) (NCT00565812)

The A6171016 or iTIC study (iNOS Trial to Investigate Chondroprotection) was sponsored by Pfizer and targeted persons with medial tibiofemoral OA [46, 47]. The efficacy of SD-6010 was evaluated by radiography using joint space narrowing in the medial tibiofemoral compartment of the study knee as the primary endpoint. A total of 1400 persons were enrolled in the main cohort (Xray + Outcome Measures), and 100 persons were enrolled in an MRI sub-cohort (patients who underwent an MRI of the knee); blood and urine samples were also collected from the small MRI sub-cohort. The duration of the trial for individual participants was 22 months.

#### Oral calcitonin (Novartis/Nordic Biosciences) (NCT00486434 and NCT00704847 — Phase 3)

Two randomized, double-blind, multi-center, and placebo-controlled trials (CSMC021C2301 and CSMC021C2302) evaluated the efficacy and safety of oral salmon calcitonin (sCT) formulated with a 5-CNAC carrier (a molecule based on Eligen® technology) in patients with painful knee OA with structural manifestations, enrolling (from June 2008–June 2011) 1176 and 1030 patients, respectively [48]. Study subjects were randomized (1:1) to oral sCT 0.8 mg twice daily or placebo (PBO) for 24 months. The primary efficacy objectives were to examine the treatment effect compared to placebo on change over 24 months in joint space width (JSW) in the signal knee measured by X-ray and to examine the change in pain and function using the Western Ontario and McMaster Universities Osteoarthritis (WOMAC) questionnaire. The eligibility criteria included persons with a current level of pain (outside of acute flare) in at least one (designated the index) knee characterized by a WOMAC Pain subscale score of between 6 and 12, inclusive (where the range of subscores is 0-20); and X-ray confirmed knee OA (KLG 2 or 3). The MRIs were obtained on a low-field scanner with pulse sequences inappropriate for further MRI evaluation in the PROGRESS OA study.

#### Vitamin D (VIDEO-Arden) Study (ISRCTN 94818153)

The VIDEO study was designed as a double-blind, randomized, and placebo-controlled trial to evaluate the effect of vitamin D supplementation on the rate of knee OA progression [49]. Four hundred and seventy-four patients aged > 50 years, with knee pain and radiographically confirmed knee OA, were randomized to receive either a placebo or 800 IU cholecalciferol daily. Outcomes were assessed at 12, 24, and 36 months. The study’s primary outcome was the difference in the rate of medial joint space narrowing (JSN) between the groups, and secondary outcomes included changes in lateral JSN, KLG, WOMAC pain, function, stiffness, and the Get up and Go test. MRI with gadolinium enhancement was further performed in a subset of patients (*n*=150). Plasma, serum, and urine have also been collected at multiple timepoints (0, 3, 6, 12, 24, and 36 months).

#### Strontium ranelate (Servier) (ISRCTN41323372 — Phase 3)

The aim of this 3-year multicenter, double-blind, randomized, and placebo-controlled trial — Strontium ranelate Efficacy in Knee Osteoarthritis triAl (SEKOIA) — was to evaluate the effect of strontium ranelate on radiological and clinical progression of knee OA [50]. The study included Caucasian ambulatory men and women aged ≥50 years with knee OA according to American College of Rheumatology (ACR) criteria, with pain on at least half of the days of the previous month (intensity ≥40 mm on a 100-mm visual analogue scale). On radiography, the included patients were KLG 2 or 3 and had joint space width (JSW) of 2.5 to 5 mm with predominant knee OA of the medial tibiofemoral compartment. The trial randomly allocated 1683 patients to three treatment groups (strontium ranelate 1g [*n*=558] or 2 g/day [*n*=566] or placebo [*n*=559]). The primary endpoint was a radiographic change in JSW (medial tibiofemoral compartment) over 3 years versus placebo. Secondary endpoints included radiological progression, WOMAC score, knee pain, and urinary CTX-II levels.

#### ILLUSTRATE-K- Lutikizumab (Abbvie) *(**NCT02087904**)*

This is a Phase 2a, multicenter, randomized, and placebo-controlled trial to evaluate the efficacy and safety of Litikizumab, an anti-interleukin-1α/β (anti-IL-1α/β), in patients with symptomatic, radiographic, and inflammatory knee osteoarthritis [51]. The study included 350 knee OA participants with KLG 2 or 3 and synovitis on MRI or ultrasound. They were randomized to receive a placebo or lutikizumab 25, 100, or 200 mg subcutaneously every 2 weeks for 50 weeks. The coprimary endpoints were a change in WOMAC pain score at week 16 and a change in synovitis at week 26.

#### ROCCELLA (Galapagos)* (**NCT03595618**)*

ROCELLA is a Phase 2, multicenter, randomized, placebo-controlled trial to evaluate the efficacy of the ADAMTS-5 inhibitor S201086/GLPG1972 in slowing cartilage loss in knee osteoarthritis. The study included 932 participants with predominantly medial knee OA KLG 2 or 3, medial femorotibial joint space narrowing grades 1 or 2, and moderate to severe baseline pain. They were randomized into one of four groups: 75, 150, or 300 mg of S201086/GLPG1972, or placebo orally, once daily. The primary endpoint was the central medial femorotibial compartment cartilage thickness change on MRI at week 52. Secondary endpoints included other structural outcomes and patient-reported outcomes.

#### Statistical analysis plan

The primary outcome is radiographic OA disease progression as defined by JSN ≥ 0.70 mm from baseline to up to 36 months follow-up. The secondary outcome is clinical symptomatic OA disease progression as defined by an increase in WOMAC Pain score by ≥ 9 points ([0–100] scale) from baseline to up to 36 months follow-up. Five exploratory outcomes will be assessed: (1) A composite outcome of radiographic and clinical OA progression as defined as meeting both radiographic and clinical OA disease definitions, (2) radiographic OA disease progression as defined by JSN ≥ 0.50 mm from baseline to up to 36 months follow-up, (3) A composite outcome of virtual knee replacement [52] defined as WOMAC Pain ([0-100] scale) + WOMAC Function ([0–100] scale) ≥ 80 points for at least 2 consecutive visits, (4) a composite outcome of virtual knee replacement as defined previously plus JSN ≥ 0.50 mm, (5) clinical symptomatic OA disease progression as defined by an increase in WOMAC Function score by ≥ 9 points ([0–100] scale) from baseline to up to 36 months follow-up.

We will use logistic regression to assess the bivariate association between each biomarker and disease progression. Associations will be summarized with odds ratios and associated 95% confidence intervals and c-statistics. Biomarkers will be assessed prior to model development to determine the best analytical strategy (e.g., log-transformation, unit-normal transformation). Each biomarker will be graphically assessed to determine suitability in logistic regression modeling (i.e., log-odd linearity assumption is held). A biomarker will be selected for multi-variable model inclusion using a liberal significance criterion (*α* = 0.20) [53].

As there is little overlap in the studies available for these analyses with regard to biochemical and MRI imaging biomarkers, separate models will be built for biochemical, MRI, and x-ray biomarkers. All selected biomarkers identified during screening will be assessed in one single multi-variable logistic regression model. A one-step, backward selection will be used to remove biomarkers not meeting the nominal inclusion criterion of *α* ≤ 0.05. Statistical interactions between predictors and collinearity will be assessed during the model-building process.

Model discrimination will be assessed with the area under the receiver operating characteristic curve (AUC). Ten-fold cross-validation and bootstrapping will be used to estimate out-of-sample performance [54, 55]. Calibration will be assessed graphically with calibration plots, calibration-in-the-large, and calibration slope [56].

Separate multivariable prediction models will be constructed for baseline biomarkers (bl-PROG) and short-term change in biomarkers (change-PROG). Multivariable prediction models will also be constructed for MRI and TBT biomarkers in combination and biochemical and TBT biomarkers in combination as the TBT radiographic measure is available for all subjects.

Alternative analytic approaches may be employed as secondary analysis including LASSO and ridge regression in the case of extremely high collinearity and restricted cubic splines to model non-linear associations between biomarker and progression. A complete statistical analysis plan is available upon request.

#### Sample size and power

We assumed that approximately 300 participants would contribute MRI biomarker data and approximately 1000 would contribute biochemical biomarker data. Power depends on the sample size, the prevalence of the outcome, and the association between biomarker and outcome. Power was estimated, presuming a true underlying increase in odds of having an outcome (i.e., progressing) for a normalized one standard deviation (1 SD) unit change in biomarker. For MRI biomarkers, a sample size of 300 affords >80% power for ORs of approximately 1.7 and larger. For biochemical biomarkers, a sample size of 1000 affords >80% power for ORs of approximately 1.4 and larger. Selected scenarios are listed in Table 7. A larger sample size or higher outcome prevalence will allow the detection of smaller ORs.

**Table 7:** Estimated statistical power

**Sample Size**	**Prevalence of progression outcome**	**Odds ratio for each 1 SD increase in biomarker**	**Estimated power**
300	11%	1.5	55.3%
300	11%	1.7	79.1%
300	15%	1.5	66%
300	15%	1.7	87.7%
1000	11%	1.4	88.4%
1000	11%	1.5	96.9%
1000	15%	1.3	79.7%
1000	15%	1.4	94.8%

#### FDA qualification and similar precedents

The FDA released a draft OA Guidance for Industry in July 2018 that recognizes OA as a serious disease and acknowledges difficulties in developing drugs for OA due to the lack of structural endpoints that translate into a clinically meaningful benefit to patients [15, 57]. The guidance also describes the FDA’s willingness to engage in discussions among regulators, sponsors of medical products, academics, and other key stakeholders to better address these gaps. To support greater engagement and alliance with regulators, the FDA has established a formal process, the Biomarker Qualification Program (BQP), to work with disease experts and stakeholders to develop, validate, and qualify measurable and reliable biomarkers (or endpoints) for specific use in drug development. The PROGRESS OA Project is directly engaged in this process with the primary goal of qualifying structural imaging and biochemical biomarkers under the described COU. Once qualified, a biomarker can be used under its qualified COU during the development of any candidate drug.

Highlighting the potential of qualification, the FNIH BC has established a precedent of success through this process by leveraging a multi-stakeholder team to qualify a composite measure of six urine biomarkers that change in response to drug-induced kidney injury before irreversible damage and earlier than traditional biomarkers. This composite measure is qualified through the BQP and can aid in detecting acute kidney injury in healthy volunteers during early-phase clinical trials. This will help improve the development of safe and effective medicines where concern has been raised that an investigational drug may cause kidney injury [58]. We intend to use similar methods in the OA space as to those used for kidney safety.

#### OA community activities to facilitate qualification

**Fig. 2:**
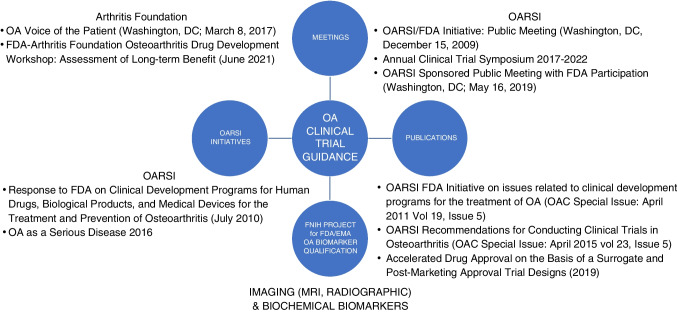
OA Community activities to facilitate qualification

Over the last decade, a series of meetings, initiatives, and publications have been convened and produced to advance the development of treatments for OA (Fig. 2). In 2007, OARSI answered a call of the FDA to provide expert advice on means of facilitating OA drug discovery and development programs; this culminated in a public presentation to the FDA in 2009 and the publication of a special issue of Osteoarthritis & Cartilage in 2011 summarizing recommendations related to trial methodology, responsiveness and reliability of patient-reported outcomes, MRI and radiographic imaging, and biochemical markers [59]. The work over the years since then has focused on maintaining communication and interaction of OARSI with regulatory agencies, pharmaceutical industry partners, and patient-oriented organizations, chief among them the Arthritis Foundation. Together, we have raised the consciousness of OA as a serious disease by producing an OARSI white paper on the subject [60]. The work has also focused on standardizing and refining OA clinical trial measures and culminated in an OARSI-sponsored initiative that led to the publication of a special issue of Osteoarthritis & Cartilage related to comprehensive recommendations for conducting Clinical trials in OA [61].

In 2017, the Arthritis Foundation brought the voice of the OA patient to the ear of the FDA in attendance at a symposium on “OA Voice of the Patient” held in Washington, DC (March 8, 2017) [62]. In 2019, OARSI sponsored a public meeting with FDA participation to highlight the tremendous unmet need for new therapies to treat OA and the current level of evidence supporting surrogacy of imaging and biochemical markers for patient outcomes (how a person/joint feels, functions, or survives). Since 2017, OARSI has sponsored an annual workshop on aspects of OA clinical trials. These workshops have engaged OA academic and pharmaceutical researchers and regulatory agency representatives in elevated discussions and conversations to advance our understanding and improve our conduct of OA trials. Topics covered have included numerous aspects and current and ongoing challenges in the OA field, such as the following: How do patient-reported outcomes fit into modern clinical development? the placebo effect in OA trials and how to reduce it in clinical studies, the regulatory side of biomarkers — implementation in clinical studies and use for trial enrichment of targeted OA endotypes, the use of biomarkers for accelerated drug approval according to subpart H and implications for this approval pathway in the conduct of OA trials [63].

In 2012, the FNIH OA Biomarkers Consortium project was initiated with the goal of formally qualifying biomarkers for prognostic indications. As described in this review, the Phase 1 FNIH study identified a subset of the most informative prognostic biomarkers, which have been advanced to Phase 2 (the PROGRESS OA study), involving qualification in the context of the placebo arms of several completed OA randomized clinical trials. FDA scientists have engaged in this endpoint debate, recently contributing a proposal for a composite endpoint of “time to total knee replacement (TKR) or severe pain or severely impaired functioning” to reduce sample sizes needed to show a drug effect compared to the use of TKR alone [64]. This widespread collaboration of all stakeholders across the field over the last decade of diligent work has created the possibility of highly anticipated breakthroughs for the development and approval of drugs to make a significant difference in the lives of those suffering from this most prevalent arthritis.

#### Next steps

The most immediate next step is to complete the analyses described above, which we aim to have completed in the first quarter of 2023. Upon completing that task, we will make the data from the analyses broadly and publicly available and publish relevant results by the investigators in collaboration with the project teams promptly thereafter. Following this, we will pursue biomarker qualification with both FDA and EMA. In parallel with these activities, we will pursue the qualification of markers through the Biomarker Qualification Program. Upon completing these activities, we will widely disseminate this information and promote the use of these biomarkers to enhance clinical trial efficiency.

## Conclusion

There are numerous unmet needs in the field of osteoarthritis. Foremost among them are the unmet needs of pain and the development of disease-modifying therapy. This project is focused on facilitating the qualification of markers for a prognostic context of use to enhance the efficiency of disease-modifying osteoarthritis trials. The field recognizes the limitations of the current regulatory standards and the primary focus of this activity is to overcome those obstacles.
